# Editorial: Obesity and diabetes: implications for brain-immunometabolism, volume II

**DOI:** 10.3389/fnins.2023.1244064

**Published:** 2023-10-03

**Authors:** Joana M. Gaspar, Alexandra Latini, João M. N. Duarte

**Affiliations:** ^1^Laboratory of Neuroimmune-Metabolism, Federal University of Santa Catarina, Florianópolis, Brazil; ^2^Graduate Program in Biochemistry, Federal University of Santa Catarina, Florianopolis, Brazil; ^3^Laboratório de Bioenergética e Estresse Oxidativo-LABOX, Departamento de Bioquímica, Centro de Ciências Biológicas, Universidade Federal de Santa Catarina, Florianópolis, Brazil; ^4^Department of Experimental Medical Science, Faculty of Medicine, Lund University, Lund, Sweden; ^5^Wallenberg Centre for Molecular Medicine, Lund University, Lund, Sweden

**Keywords:** obesity, Neuroinflammation, metabolic disorders, carnosin, COVID, mitochondrial, mitochondrial dysfunction

Globally, it is estimated that a total of 5 million deaths are related with obesity, and 1.4 million are related with type 2 diabetes *mellitus* (T2DM) (Chew et al., 2023). Such metabolic disorders predispose for numerous comorbidities, including cognitive impairment and dementia (Schmitt and Gaspar, 2023). In volume I of the Research Topic “*Obesity and Diabetes: Implications for Brain-Immunometabolism*,” we provided an update on some critical aspects of neurometabolic changes in the central nervous system (CNS) induced by inflammation associated with obesity and T2DM (Gaspar et al., 2020). In volume II of the Research Topic, we provide further understanding of mechanisms by which metabolic disease impacts the CNS, particularly brain regions involved in memory and cognition.

Zhang et al. reviewed how exaggerated feeding and obesity impact the brain through mechanisms involving insulin resistance, gut-brain axis, and neuroinflammation. In this line, the study by Yang et al. proposes that hyperinsulinemia in a model of diet-induced obesity alters mitochondrial dynamics and metabolism in microglia. This study has two sections, a study *in vivo* in high-fat diet (HFD)-fed mice, and a study *in vitro* using the BV2 microglia cell line as well as CD11b^+^ cells isolated from dissociated mouse brains. In HFD-fed mice, this study confirmed that hyperinsulinemia is accompanied by increased cerebrospinal fluid (CSF) insulin concentration and impaired central insulin signaling, and reports systemic inflammation and, in particular, hippocampal microgliosis and overexpression of IL-1β, TNF-α, CD68 and IL-16. The authors postulate that insulin mediates neuroinflammation during HFD feeding, which was tested *in vitro*, and is responsible for the known HFD-induced memory impairment (Garcia-Serrano and Duarte, 2020). It is interesting to note that these findings on HFD-induced hippocampal neuroinflammation contrast with other studies using a composition-matched control diet, in which obesity leads to memory impairment without overt neuroinflammation (e.g., Duarte et al.; Garcia-Serrano et al., 2022). Control mice in the study by Yang et al. received a regular chow, in which the content of dietary ingredients differ from that in HFD. In the second part of the study, BV2 microglia exposed to insulin showed increased proliferation and overexpression of cytokines (the later confirmed in mouse microglia *in vitro*). This inflammatory profile was accompanied by reduced cellular ATP levels, suggesting altered metabolism. Mitochondrial metabolism was not dissected in this study. Instead, the authors report reduced levels of mitofusin-2, and increased dynamin-related protein 1 (DRP1) and DRP1 phosphorylation, suggesting mitochondrial dynamics unbalanced toward increased fission. Interestingly, the authors also briefly address insulin-dependent translocation of GLUT4 in BV2 microglia, a feature that in the CNS has been thought to be exclusive of neurons (reviewed in Duarte, 2023).

Campillo et al. employed quantitative magnetic resonance imaging (MRI) approaches for examining HFD-induced neuroinflammation. Brain areas of focus included those involved in energy homeostasis and appetite control (hypothalamus, nucleus accumbens, infralimbic area), and also the hippocampus, which controls spatial memory and is impaired after HFD feeding (Garcia-Serrano and Duarte, 2020). This study is particularly interesting for its longitudinal design, across metabolic syndrome development, and has included mice of both sexes. While male mice rapidly developed obesity paralleled by fast increases in magnetization transfer ratio, females showed delayed obesity and no MRI-signs of neuroinflammation. The study included analyses of metabolite profiles, revealing HFD-induced brain metabolite alterations in females larger than males. Noteworthy, metabolite levels were reported as ratio to total creatine levels, which in turn can be modified by diet-induced obesity (Garcia-Serrano et al., 2022). Therefore, calculates ratios might mask metabolite alterations in the study by Campillo et al.. Nevertheless, altogether, these results show that the faster metabolic syndrome development in male than female mice is paralleled in either sex by specific CNS alterations detectable MRI. The translatability potential of this research is enormous.

Not only unhealthy food choices drive metabolic disease with impact on the CNS, the impact of sedentary lifestyles needs to be accounted for. In this Research Topic, Shao et al. reviewed the literature supporting the impact of physical activity and exercise on cognition, and brain structure and brain activity measured by MRI in obese children. Despite abundant controversy, this review contributes to shed some light on the beneficial effects of long-term exercise on brain structural and functional integrity in overweight children, and points at research directions to fill in knowledge gaps.

This Research Topic has also included an article that addressed the particular severity of COVID-19 infection among individuals with obesity and/or metabolic disease, and proposed an adjuvant therapy for the viral infection in such conditions (Diniz et al.). Diniz et al. reviewed metabolism of carnosine, the use of carnosine metabolites as predictive of morbidity or mortality in metabolic disease and diabetes, especially in patients affected by viral infections by COVID, and the potential use of carnosine as therapeutic approach in diabetes and other afflictions reported in long COVID patients.

Finally, Perez-Bonilla et al. investigated the role of Neurotensin Receptor-1 (NtsR1) on appetite regulation and weight gain control. Following previous work locating NtsR1 expression in dopaminergic neurons of the ventral tegmental area (VTA), Perez-Bonilla et al. developed a mouse line with conditional deletion of NtsR1 in dopaminergic neurons. Experiments on this model were paralleled by work in mice after viral transfection for generating an adult-onset, VTA-specific deletion of NtsR1. Altogether, the study's findings show that developmental or adult deletion of NtsR1 from dopaminergic neurons protects mice from weight gain upon HFD exposure, while feeding was only impacted by adult deletion of the receptor. While not yet allowing to fully understand neuromodulation actions of neurotensin, this work already provides cues that NtsR1 in dopaminergic neurons plays critical roles in appetite control.

In sum, this Research Topic explores mechanisms underlying effects of obesity and T2DM on cognitive performance, including both clinical and preclinical observations. Besides efforts to understand the molecular mechanisms leading to obesity-associated cognitive impairments, the Research Topic extends the discussion on preventive and therapeutic strategies for obesity and associated comorbidities, which could be implemented in educational and healthcare systems.

## Author contributions

All authors listed have made a substantial, direct, and intellectual contribution to the work and approved it for publication.
